# Replications in Comparative Cognition: What Should We Expect and How Can We Improve?

**DOI:** 10.26451/abc.07.01.02.2020

**Published:** 2020-02

**Authors:** Benjamin G. Farrar, Markus Boeckle, Nicola S. Clayton

**Affiliations:** 1 Department of Psychology, University of Cambridge, Cambridge, UK; 2 Deparmtent of Psychology and Psychodynamics, Karl Landsteiner University of Health Sciences, St. Pölten, Austria

**Keywords:** Comparative cognition, Replication, Reproducibility, Evidence

## Abstract

Direct replication studies follow an original experiment’s methods as closely as possible. They provide information about the reliability and validity of an original study’s findings. The present paper asks what comparative cognition should expect if its studies were directly replicated, and how researchers can use this information to improve the reliability of future research. Because published effect sizes are likely overestimated, comparative cognition researchers should not expect findings with *p*-values just below the significance level to replicate consistently. Nevertheless, there are several statistical and design features that can help researchers identify reliable research. However, researchers should not simply aim for maximum replicability when planning studies; comparative cognition faces strong replicability-validity and replicability-resource trade-offs. Next, the paper argues that it may not even be possible to perform truly direct replication studies in comparative cognition because of: 1) a lack of access to the species of interest; 2) real differences in animal behavior across sites; and 3) sample size constraints producing very uncertain statistical estimates, meaning that it will often not be possible to detect statistical differences between original and replication studies. These three reasons suggest that many claims in the comparative cognition literature are practically unfalsifiable, and this presents a challenge for cumulative science in comparative cognition. To address this challenge, comparative cognition can begin to formally assess the replicability of its findings, improve its statistical thinking and explore new infrastructures that can help to form a field that can create and combine the data necessary to understand how cognition evolves.

Comparative cognition is a broad field which investigates how animals acquire, process and use information (Beran et al., 2014; Shettleworth, 2009). Within the field there are several different approaches to what cognition is and how best to study it (Bayne et al., 2019; Boyle, 2019; Chittka et al., 2012; Eaton et al., 2018; Heyes, 2019; Zentall, 2018). Nevertheless, most comparative cognition research shares the goal of understanding how cognition evolves by assessing the similarities and differences in cognition across a wide range of species (MacLean et al., 2014; ManyPrimates et al., 2019a, b). Increasingly, its findings are being applied to wider issues such as conservation and animal welfare (Greggor et al., 2014; Nawroth et al., 2019).

Despite this great diversity, comparative cognition has yet to have much of an investigation into the reliability of its findings. This is surprising because across many scientific disciplines there is great concern about the reliability and reproducibility of research findings (Baker, 2016). In psychology and related fields, large-scale attempts to replicate published research have only produced significant results around 60% of the time (Camerer et al., 2016, 2018; Ebersole et al., 2016; Klein et al., 2018; Open Science Collaboration, 2015; Zwaan et al., 2018). And in fields similar to comparative cognition, some established effects have not consistently replicated, such as the blocking effect in animal learning (Maes et al., 2016) and non-verbal studies in infant theory of mind (Poulin-Dubois et al., 2018). However, we currently do not know how generalizable these results are to comparative cognition. This is further complicated as comparative cognition is a very heterogeneous field. Some areas of comparative cognition *do* bear the hallmarks of science that has proven difficult to reproduce, namely small sample sizes, noisy measurements and unlikely hypotheses (Forstmeier et al., 2017), whereas other areas may be less affected by low replicability rates due to using research designs which typically show higher replicability, such as within-subjects designs in which there are many trials for each animal tested (Smith & Little, 2018).

In light of this, the present paper focuses on two questions. First, what should comparative cognition from direct replication studies? And second, how can we use this information to understand and develop comparative cognition as a science? To address these questions, we will apply arguments that have been made across fields in response to their replication crises or “credibility revolutions” (Gelman & Geurts, 2017; Vazire, 2018). While the paper focuses on what comparative cognition should expect from replication studies, and how it can use this knowledge to improve in the future, the arguments can also be applied retrospectively to assess the evidential value of previously published findings. This is important as the established scientific system can select for unreliable and misleading research (Lazebnik, 2018; Smaldino & McElreath, 2016), yet scientific self-corretion does not happen as often as the textbook definition implies (Ioannidis, 2012; Rohrer et al., 2018).

Section 1 of this paper examines statistical cues to replicability, and specifically ask what can be gained from looking at *p*-values and effect sizes in published findings. Section 2 then asks whether the different research designs employed across comparative cognition may lead to systematic differences in replicability. Next, Section 3 makes the case that single direct replication studies will be unable to falsify many claims in comparative cognition, even if they are false. It then discusses the difficulties that comparative cognition will face when performing and interpreting the results of replication studies. Finally, Section 4 takes a more positive stance by highlighting steps comparative cognition can take to calibrate its expectations about replicability and improve the replicability of its own research.

## Section 1 – *p*-values, Effect Sizes and Direct Replications

Some cues about a result’s replicability can be found in statistical reports, in the reported *p*-values, effect sizes and confidence intervals. However, the statistics presented in published reports are unlikely to provide an accurate estimate of an effect size because of a publication bias favoring positive results. If papers are selected for publication based on a significance criterion such as *p* < .05, then it is more likely that studies overestimating effect sizes will be published than studies that underestimate the effects (Cumming, 2008; Fiedler & Prager, 2018; Gelman, 2018b; Hedges, 1984; Lane & Dunlap, 1978; Piper et al., 2019; Vasishth et al., 2018). In cases where many low powered studies are performed, as we might expect in comparative cognition, effect sizes can be overestimated upwards of 100%, and the likelihood of an exact replication study producing a significant effect in the same direction as the original study can be low, as our later simulation studies will show. But first, to illustrate this descriptively, we now discuss three hypothetical scenarios of a direct replication study, in which the original study estimated the unknown true effect size either exactly, underestimated it or overestimated it. These direct replication studies have the same sample size as the original study and sample from the same population.

### Scenario 1 – Original Study Estimated the Real Effect Size Exactly

If an original study estimated the real effect size for that design exactly, then the likelihood of the replication study to return a positive result is the power of the statistical test to detect this effect size. Therefore, if the original study returned a positive result that was just statistically significant, such as *p* = .049, then the likelihood of an exact replication study returning a positive result is approximately 50%; like tossing a coin (Piper et al., 2019). This is because, 50% of the time the replication study will overestimate the effect size, resulting in *p* < .049, whereas 50% of the time it will underestimate the effect size, resulting in *p* > .049, and this is non-significant with an alpha of .05. However, as the *p*-value of the original study decreases, then ceteris paribus, the chances of a positive replication result increase.

### Scenario 2 – Original Study Underestimated the Real Effect Size

If the original study underestimated the real effect size, then it becomes more likely that a direct replication study of equal sample size will return a positive result as the replication study will exceed that effect size over 50% of the time. In the example of a just significant result, *p* = .049, in the original study, the likelihood of a positive direct replication with the same sample size is now greater than 50%.

### Scenario 3 – Original Study Overestimated the Real Effect Size

However, the most likely scenario is that the original study will have overestimated the real effect size, particularly for studies that produced p-values just below the alpha level. This scenario is most likely for two reasons. First, as publications are selected based on a cut-off point, studies that overestimate effect sizes are more likely to be published than studies which underestimate effect sizes (Hedges, 1984). For example, if exactly estimating the “true” effect size would have yielded a *p*-value of .06 in a design, then only samples overestimating the effect size would be published. Hence, in direct replication studies, the replication *p*-value would regress to the mean, .06, and the original study would have less than a 50% chance of a positive direct replication with the same sample size. Second, the overestimation of published effect sizes is further exacerbated if research practices that produce more significant results are used, which appears relatively common (Fraser et al., 2018; John et al., 2012; Simmons et al., 2011). Hence, the default expectation when directly replicating a study with a *p*-value in the range of .01 < *p* < .05 is that there is a good possibility that a direct replication study with the same sample size will not produce a statistically significant result in the same direction as the original study, even if there is a real underlying effect.

#### Section 1.1 – Simulation Study

Another way to view these ideas is through simulation studies, and to this end we simulated a very simple model of comparative cognition research (for details and code see the Appendix). First, we simulated 40,000 studies, comprising of four sets of 10,000 studies with a power of 80%, 50%, 20% and 5% (i.e., false positive results only in the 5% group) to detect a known difference between two groups of 10 animals. The groups were compared using a one-tailed two-sample *t*-test and results with *p* < .05 were termed significant and “published.” The results of these hypothetical studies are displayed in Table 1, along with a comparison of the specified and “published” effect sizes.

In total, 15,471 of the 40,000 simulated studies produced significant results, with approximately half of these from the 80% powered designs (Table 1). However, even when running studies with 80% power, the average effect size was inflated as only the significant studies were published. As the power of the tests decreased, the degree of inflation increased. Next, we derived the expected proportion of direct replication studies that would return a significant result if all of the 15,471 published studies from the simulation were replicated exactly. Again, these exact replications had equal sample size to the original studies, and the results are broken down across different ranges of the published *p*-values in Table 2. The overall expected replication rate of this group of research, conducted with a quarter of studies having 80%, 50%, 20% and 5% power was 0.60.

These results show that the expected replication rate of published research from this model can be close to 0.5, or even below 0.5, for just significant results. Notably, these results are in the absence of any false positive inflating research practices (Fraser et al., 2018; John et al., 2012; Simmons et al., 2011); they are solely the consequence of only publishing research with *p* < .05 and performing at least some research with relatively low power. However, this simulation does not accurately characterize the field of comparative cognition: Not all comparative cognition research is performed with 80%, 50%, 20% or 5% power, or using two-sample t-tests comparing two groups of 10 animals. As such the numbers should not be taken as a literal estimate of the replicability of comparative cognition research; rather the simulation study shows that the research and publication methods used in comparative cognition do lead to effect sizes being over-estimated and a replication success likely to be closer to 50% than 100% for just significant published findings. Conversely, experiments reporting very low *p*-values, with associated effect sizes with confidence intervals that are narrow and far from 0 can be good indicators of a reliable statistical effect. Finally, one scenario in which these conclusions might be inappropriate is when considering research using designs and test combinations in which the *p*-value distribution is not uniform or near uniform under the null hypothesis, such as with a binomial test and a small number of observations.

*Conclusion 1: Comparative cognition should not expect just significant initial findings to replicate consistently, and published effect sizes are likely to be overestimated because of a publication bias towards positive results*.

Section 2 now focuses on whether the differences in research methods across comparative cognition, that were not considered in the simulation study, can allow us to estimate the replicability of research in comparative cognition.

## Section 2 – Replications Across Comparative Cognition: What can we Expect?

Section 1 showed that, all else being equal, studies in comparative cognition with lower *p*-values are more likely to replicate than studies with higher *p*-values, although the *p*-value is only a weak predictor of replication success (Cumming, 2008). However, this begs the question: which features of comparative cognition research lead to effect sizes large enough to be reliably detected, and thus produce low *p*-values and successful replication studies?^1^

One of the areas in which comparative cognition research methods differ markedly is in the number of trials they use, for example experiments using touchscreens and short time periods can use hundreds or thousands of trials (e.g., Beran, 2006) whereas experiments using more constrained behaviors such as food caching usually use considerably fewer trials (e.g., Ostojić et al., 2017). Given that sample sizes are often small in comparative cognition, many “effects” might be too small to detect on a single trial, as the errors associated with measuring these behaviors on a single trial are large. Having many trials in within-subjects designs can address this issue by enabling more precise estimation and consequently smaller *p*-values and more replicable results to manifest. All things being equal, within-subject designs are more powerful to detect effects than between-subject designs with similar resources, and studies with more trials will be more likely to detect effects than those with few trials. This tendency of within-subject design with many trials to be more replicable is visible in the human replication literature (Open Science Collaboration, 2015; Zwaan et al., 2018). Many reliable findings in comparative cognition will have harnessed this ability to perform repeated tests on individual animals that are often highly trained on the task, although whether a certain number of trials are “sufficient” or not will depend on particular features of the task at hand, notably the size of the effect and the measurement error.

*Conclusion 2: Studies using within-subject designs with many trials and less-noisy behaviors are more likely to produce replicable results than studies with similar resources that use between-subjects designs, within-subject designs with few trials or more-noisy behaviors*.

To illustrate this point in an applied setting, we performed another simulation based on a real experiment. Emery and Bird (2010) reported that seven rooks looked longer at two images of physically impossible events than two images of physically possible events. On average, the rooks looked for around 1000ms at each image, and approximately 200ms longer at the physically impossible events than the physically possible ones (data from Emery & Bird, 2010: Experiment 1).

**Figure 1:**
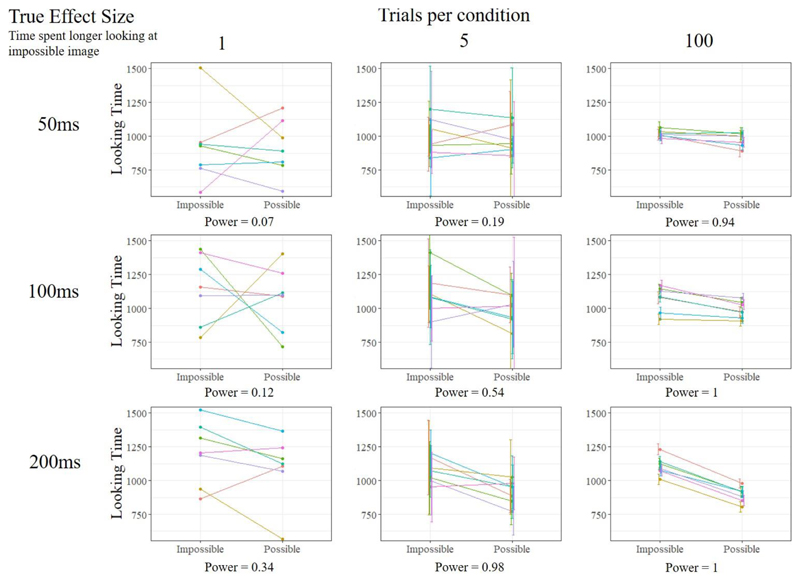
Graphs showing example data from nine different designs of a looking-time experiment in rooks. These designs vary on the underlying effect size (50 ms, 100 ms, 200 ms), and the number of trials per condition (1, 5, 100). *N* = 7 for all designs. The power of each design is printed below each graph and was calculated by simulating 10,000 studies in each design and calculating the proportion of *p*-values less than .05.

To investigate the role of trial number and effect size on the replicability of significant results in experiments like this, we simulated data for a series of hypothetical experiments, in which seven rooks were given either 1, 5 or 100 trials in each condition, and where the average effect size was either 200 ms, 100 ms or 50 ms. We decided that 200 ms is likely quite a large effect, as it was detected in a sample size of 7 from two trials per condition, and so also included two smaller effects in the simulation. For each design we ran 10,000 simulations to estimate the power of the design and test combination. In the data simulation, we included a fixed main effect of condition, random intercepts and slopes for each individual, and a small random intercept for the stimuli (See Appendix). Figure 1 plots an example of the data from one of these experiments, and the power for each design is presented under each graph.

Figure 1 shows that both increasing trial number and studying larger effects can lead to large increases in power, even with a constant sample size. When there was only 1 trial per condition (leftmost panels), the power of the design to detect the whole range of effect sizes was low. Notably, this design was prone to producing results *in the opposite direction* to the true effect (see Gelman & Carlin, 2014). For the smallest effect size, 50 ms, 32% of single trial experiments led to effects estimated in the opposite direction to the true effect, and 13% of the significant results were in the opposite direction to the true effect. This design was completely insensitive to individual differences in the effect, and the significant results from these studies, if published, would lead to large overestimations of the true effect size and exact replication studies often would return non-Signiant results, at the rate of 1 – power (as per the results of the simulation in Section 1). One striking aspect of Figure 1 is just how misleading visualizations of single trial studies can be. As the uncertainty is never measured for each bird in each condition, we cannot include this on the visualization, resulting in a plot that looks clean but gives us little information about the uncertainty of our estimates.

The benefits of increasing trial number are visible when there were 5 trials per condition (middle panels). At the largest effect size, the design would nearly always detect the main effect of condition. However, this design is still quite insensitive to the real individual differences we included in the simulation, and exact replication studies would still only return significant results around 50% of the time for the 100 ms effect. This contrasts with the 100 trials per condition design (rightmost panels), which, in addition to detecting even the smallest effect very reliably, was also sensitive to many of the individual differences between animals within each study simulation. Studies using such designs in comparative cognition can produce, and in many areas already do produce, very replicable results.

However, this benefit of running many trials across participants may not be accessible for some areas of comparative cognition. This is the case when theoretical constraints restrict experimental designs to using a single test trial, in order to avoid experimental confounds. For example, in some memory tests the animals are tested in extinction, to prevent cues from the to-be-remembered items directly influencing their searching or responding behavior (Adams & Dickinson, 1981; Clayton & Dickinson, 1998). Such studies cannot use many test trials in within-subjects designs because this would invalidate the test itself; for example, the animals could learn that no food is provided in the test trials and therefore that there is no value in searching for the food. Hence, for some hypotheses in comparative cognition, the optimal methodological design may not be one that produces highly replicable results, if replicability is measured by statistical significance only. Nevertheless, all else being equal, studies employing many trials will produce more replicable results than studies using few trials. However, this does not mean that most many-trial studies and no few-trial studies will replicate successfully. Further, even if this were true, it does not mean that many trial studies are always “better”. As ever, there are trade-offs between likely replicability, validity and resource investment. Many experiments that use many trials, such as reaction time studies in humans, can have participants perform hundreds of trials in minutes, whereas in many animal studies this is not possible. The reliability-resource trade-off is just that: a trade-off, and researchers should seek to evaluate and improve reliability with this in mind.

*Conclusion 3: Some areas of comparative cognition face replicability/validity and replicability/resource trade-offs*.

### Section 2.1 – Replications Across Comparative Cognition: Small-N, Many Replicates

One research area that consistently uses many trials to produce replicable results is small-N research. Small-N research capitalises on the benefits of using many trials on each individual subject, yet rather than seek to estimate population parameters they treat the individual as the replication unit (Little & Smith, 2018; Smith & Little, 2018). Each individual is its own experiment, and experiments are thus replicated by using more than one individual. Smith and Little (2018) explain this approach:

We argue that some of the most robust, valuable, and enduring findings in psychology were obtained, not using statistical inference on large samples, but using small-N designs in which a large number of observations are made on a relatively small number of experimental participants. We argue that, if psychology is to be a mature quantitative science, its primary theoretical aim should be to investigate systematic, functional relationships as they are manifested at the individual participant level. The estimation of population parameters, while not unimportant, is arguably of secondary concern and should probably be investigated using more refined techniques for characterizing individual differences than the blunt instrument of simple averaging that conventional statistical methods provide. (p. 2084)

The small-N approach is found across psychological research, for example in Ebbinghaus’ forgetting curve and many psychophysical experiments. And interesting hypotheses are often tested at the individual level in comparative cognition - think of studies of number comprehension in Alex the parrot (Pepperberg & Gordon, 2005), of fast mapping with Chaser the dog (Kaminski et al., 2004) and of working memory in Ai and Ayumu (Inoue & Matsuzawa, 2007). Individual-level data are also widely used in comparative psychology. Learning curves, for example, are usually most informative when plotted at the individual level, and this is part of the reason why Skinner rejected null-hypothesis significance testing (Gigerenzer et al., 2004; Skinner, 1956). Small-N research and individual-level data are particularly informative when we can be confident that the results will generalize to at least some more individuals. For example, understanding the learning mechanisms of Lab Rat 377 logically will inform us about rat learning more generally, and hence a small-N approach might be appropriate here. However, while small-N research has a formal approach to inference at the individual level (Smith & Little, 2018), there is no formal method of generalizability. In comparative cognition, when strong evidence is found at the individual level, the question “will it replicate?” asks “how will it generalize to similar individuals who have had similar experiences?” In contrast, group-level comparative cognition research, the question “will it replicate?” may be more synonymous to “is it a reliable statistical effect in the given population?” Notably, the choice between a small-N and “large-N” design is not dichotomous. Large-N studies in comparative cognition can, and do, capitalize on the benefits of small-N designs. Large-N research can make both individual level and population level inferences without large costs. In performing high powered tests at the individual level, this also increases the power for any population level inferences made.

Therefore, the small-N vs large-N distinction may be somewhat misleading when discussing replicability and could instead be replaced by many vs. few trials. It is misleading to say that the researchers’ hypothesis level (individual vs population) changes something general about the replicability of the data they produce. But importantly, if comparative cognition researchers wish to make statistical inferences at the population level in order to address evolutionary questions, then group level analyses are necessary. However, the accuracy of these population inferences will be limited by the representativeness animals studied with respect to the target population or species in general. If we cannot be confident that the samples we use are representative of the populations we wish to make inferences about, then we should be cautious in how we use their data in evolutionary models. Moreover, it is certainly possible to qualitatively address evolutionary questions with individual level data, particularly when individual level data offer convincing existence proof of what certain species are capable of. For example, when Megan passed the trap tube task (Povinelli, 2003), this was overwhelming evidence that chimpanzees are in theory capable of learning how to pass this particular form of the task. Note, the evidence is only overwhelming because of how inconsistent Megan’s behavior was with her not avoiding the trap, i.e., a *p*-value of .000000000135 (80/100 correct trials, with a null hypothesis as 50/100).

*Conclusion 4: Different research approaches have different meanings by replicability (see Lazic et al., 2018). While these approaches answer different questions, comparative cognition can benefit from both approaches, and can often employ both simultaneously*.

### Section 2.2 – The Data are not Everything

Thus far, this paper has considered statistical and design features of replicability. However, there is much more information about replicability than can be gleaned from reported statistics alone. When Daryl Bem provided evidence for a physically impossible skill, precognition, across nine studies, statistical markers were part of the subsequent refutation (Francis, 2012; Schimmack, 2012; Wagenmakers et al., 2011). However, it was the sheer implausibility of the effect that provided the clearest indicator that it would not replicate (Chambers, 2017). This ability of researchers to detect findings that are likely unreliable extends from the physically impossible to more plausible research results too. Research using forecasting and prediction markets has shown that groups of experts, on average, can be surprisingly accurate at identifying research that will not replicate (Dreber et al., 2015; Forsell et al., 2018) – and there is no reason why this should be any different in comparative cognition. If your belief in something is very low, then a just-significant result should not affect this belief greatly. This is not to say that researchers should be insensitive to evidence, but they should critically assess whether there is sufficient evidence that a given statistical effect is accurate, and whether this statistical effect is strong evidence of the authors’ claims. In particular, extra-ordinary claims require extra-ordinary evidence, and this will usually not be provided by single studies with just-significant results, or even a set of such studies.

*Conclusion 5: The data aren’t the full story – aggregated expert beliefs about replicability might be accurate, whatever they are based on*.

## Section 3 – Difficulty in Performing and Interpreting Replication Studies in Comparative Cognition

Given that many research findings might be unreliable in comparative cognition, a next step could be to call for a suite of systematic, direct replications studies to identify findings that are robust, which would enable researchers to perform meaningful meta-analyses on rich datasets with low bias. While we believe there are strong reasons to support such claims (e.g., see Beran, 2018; Stevens, 2017, although neither make such a strong claim), there are many valid barriers to performing and interpreting replication studies in comparative cognition that should be considered. These barriers mean that it may not be possible to perform truly direct replication studies in comparative cognition, because:

- Restricted resources mean that it is not possible to directly replicate some findings
- Statistical estimates from both original and replication studies will be too noisy to be able to detect differences between them with confidence
- There will be real and often large differences between animal behavior in original and replication studies

### Section 3.1 - Restricted Resources Mean that It is not Possible to Directly Replicate Some Findings

As comparative cognition is a small field represented by many different research questions in many different species (Beran et al., 2014; Shettleworth, 2009), when a laboratory stops working on a certain species the likelihood of direct replication studies of these results in the near future approaches zero. For example, our Comparative Cognition laboratory in Cambridge performed a series of studies on cache-protection strategies in California scrub-jays (*Aphelocoma californica*) which it no longer houses^2^. Consequently, the possibility of these studies being directly replicated with California scrub-jays in this lab in the coming years is very low. Multi-lab collaborations could help, for example there is one laboratory that currently publishes on caching in California scrub jays (Clary et al., 2019), however such collaborations will be difficult to implement without a change in incentive structure and attitude towards replication studies (ManyPrimates et al., 2019b; Nosek et al., 2012).

Furthermore, the small and specialist structure of comparative cognition means that often it will be very difficult for people outside of a few laboratories to probe the reliability of findings through direct replication studies. Comparative cognition researchers engaging in empirical work should recognize this privileged position, invite dissenters to collaborate with them, and embrace the challenge to produce reliable research findings – as often others will not be able to help. Yet while researchers should take on the responsibility for estimating and justifying the reliability and accuracy of their own results, this also requires endorsement from, and collaboration with, other researchers, funding bodies and editors and journals.

*Conclusion 6: Often, direct replications will not be immediately possible due to a lack of resources and researchers should explore collaborative methods to estimate the reliability of their research*.

### Section 3.2 Statistical Estimates from Both Original and Replication Studies will be Too Noisy to be Able to Detect Differences Between Them with Confidence

In order to detect a difference between the results of an original study and its direct replication, only looking at the significance of the results is not appropriate. Considering statistically significant replications to be the only “successful” replication studies, and all others to be “unsuccessful”, is misleading (Gelman, 2018a) as it dichotomizes replication attempts as either successful (*p* < .05), or unsuccessful (*p* > .05). While a replication study with *p* = .049 would be considered a success, a replication study with *p* = .051 would be considered a failure. If the sample size of a replication study is not much larger than the original study, it is not surprising that many replication studies will not yield significant results, even if there is a real effect being studied (see Section 1). In fact, more liberal, and perhaps more appropriate, interpretations of large-scale replication studies results provide higher estimates of replicability than those first publicized. For example, it is often reported that the Open Science Collaboration (2015) produced only 35 positive replication results from 97 positive original findings. However, Etz and Vandekerckhove (2016) used a Bayesian analysis to suggest that “75% of [replication] studies gave qualitatively similar results [to the original studies]”, but also noted that “the majority of the studies (64%) did not provide strong evidence for either the null or the alternative hypothesis in either the original or the replication” (p. 1). Future replication projects in human psychology have attempted to address this concern by swamping the sample sizes of the original studies (e.g., Camerer et al., 2018). However, as Morey and Lakens note (2016), “sample sizes are so small in psychology that often one cannot detect even large differences between studies. High-powered replications cannot answer this problem, because the power to find differences in results from a previous study is limited by the sample size in the original study” (p. 1). When this concern is translated to comparative cognition, and its constraints on sample sizes, it is clear that producing replication studies that can assess the veracity of the original claim will be very challenging.

*Conclusion 7: Comparative cognition might not have the resources to produce highly-powered and informative replication studies of many claims*.

### Section 3.3 - There Will be Real and Often Large Differences Between Animal Behavior in Original and Replication Studies

Assuming that some form of direct replication studies are performed, interpreting their results becomes even more difficult when the many reasons why a comparative cognition replication study might fail to produce significant results are considered, even if the original effect was “true”. One example is that animal behavior often has large seasonal and developmental variation. For example, a food-caching experiment would fail to replicate outside of caching seasons, and a memory experiment performed on young animals might not replicate when the same animals are tested in their old age. Such failures to replicate could be due either to the original results being a false positive, or a well-motivated alternative hypothesis, like the memory performance of animals decreasing with age. Furthermore, the experiences of animals, particularly those that are highly trained on certain apparatuses, can prevent results from being easily replicable. This can be either in principle (e.g., some zoos might not be able to house the same equipment of laboratories), or in practice (e.g., the same equipment might have different effects in different laboratories).

*Conclusion 8: Temporal and developmental variation in animal behavior will influence the likelihood of replication success*.

Needless to say, when direct replication studies using a new sample from the same population can be performed, these will be an effective method of assessing the validity of an effect. However, in comparative cognition, it is not feasible to assume new samples can be repeatedly taken from the same population – in fact it is often unclear what “populations” we are studying in general. Rather, different groups of animals of the same species from different research sites may be best viewed as different populations with respect to many cognitive effects. In biomedical research, researchers report differences in physiology and behavior between laboratories, even when they test the exact same strain of animals with the exact same protocol (Crabbe et al., 1999). These real differences in nominally similar animals when exposed to the same treatment means that results are difficult to reproduce between-laboratories (Voelkl & Würbel, 2016)^3^, but also within laboratories when the same animals are tested repeatedly (Karp et al., 2014). That these differences are present in highly standardized conditions, such as biomedical mouse research, raises the likelihood that real and potentially large effects of site and time will reduce the replicability of animal cognition research.

A recent example in animal cognition comes from the ManyPrimates collaboration (ManyPrimates et al., 2019a, b). Here, they collected data on 176 individual primates from 12 species on a delayed response task, in which primates had to wait either 0, 15 or 30 s before choosing the location where food was hidden (ManyPrimates et al., 2019b). While there are notable similarities within and between species’ behavior across sites, some large differences can still be observed. For example, the 12 chimpanzees from the Wolfgang Köhler Primate Research Center greatly outperformed the 12 chimpanzees from Edinburgh Zoo. If such a between-site difference is present in a relatively simple and robust cognitive task, in which inter-individual differences might be expected to be low (Hedge et al., 2018), this suggests that even larger between site differences could manifest for more noisy behaviors in comparative cognition replication studies. While in the ManyPrimates data the variation within chimpanzee performance was low compared to the variation between species, and some species (e.g., capuchin monkeys) were very similar across sites, it was only possible to know this because they did sample different groups of animals from the same and different species. In contrast, multi-site studies in comparative cognition that do not sample from multiple groups of the same species risk confounding or obscuring between-species differences in behavior because they are unable to dissociate the contributions of species and site differences to the data. Even when making within-species comparisons, direct replications between sites in comparative cognition could be seen as similar to cross-cultural studies in humans, and as such lie closer to conceptual rather than direct replications. Further, if we want to replicate the between species inferences that are a cornerstone of comparative cognition, ideally new samples from new sites of each species should be recruited. This will be very difficult to achieve in practice, but researchers must recognize the uncertainty in between-species inferences based on data from single sites.

*Conclusion 9: Site specific differences in behaviors make direct replication studies sampling from new populations difficult to interpret, and could confound many between species comparisons*.

Overall, it will not be possible for researchers to identify results as “false positives” through a limited number of direct replication studies. Often, a researcher wanting to replicate a study will not have access to the desired species, and even if they did, they would be unlikely to be able to produce a result that they could confidently conclude is statistically different from the original study. Furthermore, our default assumption should be that there will be real quantitative differences between the results of replication and original studies. This holds both for effects which we might be confident are true positives, for example the observed difference between Edinburgh zoo chimpanzees and Wolfgang Köhler Primate Research Center chimpanzees in ManyPrimates, and for effects that we might be uncertain are true, such as those supported by a few just significant *p*-values. Many of the claims that are supported by a few just significant results may be practically unfalsifiable – precisely because we would not expect them to replicate consistently in similar studies even if there was a true effect. However, this does not mean that all findings reflect “true effects” in comparative cognition, instead it means that single small replication studies will not absolutely falsify a claim, and neither have many original studies proven their claims. Instead of focusing on the “presence” or “absence” of an effect in a replication study, a more fruitful approach would be to focus on whether there are meaningful differences between original and replication (Gelman, 2018a; Morey & Lakens, 2016). However, often the estimates of both the original study and the replication study will be so imprecise that we often will not have the resources to detect these differences. To account for this, comparative cognition researchers can focus on expressing the uncertainty about their findings, rather than the absolute veracity of a claim or its statistical significance. This should act prospectively when interpreting the results of replication studies and new studies, but also retrospectively for small studies that have made bold claims on the basis of weak statistical evidence.

*Conclusion 10: Single replication studies are unlikley to absolutely falsify claims. A focus on effect sizes, meaningful differences between studies and communicating uncertainty should be a long-term aim for comparative cognition research*.

## Section 4 – Recommendations for a more Reliable Comparative Cognition

While direct replication studies will often be difficult to perform and interpret in comparative cognition, they will be a useful tool to identify effects that are reliable and robust, and effects that are not. Furthermore, the results of these direct replication studies will help to calibrate researchers’ expectations about the replicability and evidential value of past research and improve the replicability of future research. If many effects do turn out to be difficult to replicate in comparative cognition, then the field could consider increasing the standards of evidence required to support a statistical claim. This could include measures to ensure the current nominal false positive rate of 5% is not greatly inflated, which can be achieved through a greater awareness of how analytical flexibility affects false positive rates (Gelman & Loken, 2013; Simmons et al., 2011). Furthermore, pre-registrations and registered reports can facilitate researchers in controlling their false positive rate by reducing unintentional biases in data collection, analysis and publication (Bishop, 2019; Chambers, 2017; Nosek et al., 2018), and open data and code can facilitate error detection and correction.

A more extreme intervention to increase the proportion of true claims in the comparative cognition literature would be to decrease the field’s false positive rate by changing the level of significance to *p* < .01 or *p* < .001 before a null hypothesis can be rejected (Benjamin et al., 2018), or by requiring that all effects are replicated directly before they can be published. While this would decrease the likelihood of misleading findings entering the literature, it would come at a large cost of either increasing the rate of false negative results or reducing the number of studies that can be performed in total. In a field where resources are limited and false negative results likely common, such a large and proscriptive shift against false positive results might have greater costs than benefits. Perhaps then it is better for individual researcher groups to set and justify the standard of evidence that they find acceptable after considering a range of questions (Lakens et al., 2018): what resources do you have? how important is the question? what are the consequences of getting the answer wrong? can you justify why you have chosen to allocate the resources you have to a particular question? And then if a result is unclear, researchers should describe their uncertainty and consider replicating the study. Embracing and describing such uncertainty is incredibly important to avoid misleading conclusions, particularly when using dichotomizing tools such as hypothesis tests (Glass, 2010; Lambdin, 2012; McShane et al., 2019). Many of the claims that will turn out to be misleading in comparative cognition will be those that have been prematurely accepted in light of weak and uncertain statistical evidence against a null hypothesis. When a just significant result is obtained, rather than claiming the presence of a real statistical effect that a theory is true, researchers should recognize they have weak evidence of a true statistical effect that *might* corroborate their theoretical claim.

In the future, researchers may consider increasing the informativeness of the evidence that they produce. To achieve this, there are a range of design factors to consider. As well as increasing the sample size, increasing the number of trials in a design can increase its power to detect theoretically important effect sizes (Rouder & Haaf, 2018), and increasing the salience of manipulations can increase the size of an underlying effect, making it easier to detect with our resources (but see Fiedler, 2011). While sample size calculations and design decisions can be difficult, there is a growing literature on power and sample size planning that can help researchers think about these issues (Gelman & Carlin, 2014; Morey, 2019; Rothman & Greenland, 2018; Rouder & Haaf, 2018). Importantly, power analyses and their analogues should not be viewed as a box-checking exercise or a golden bullet, and in many cases comparative cognition researchers could be better off performing design or sensitivity analyses based on their resource constraints. Such analyses ask, given our limited sample size, what size of effect would our study designs be able to detect, and what is the effect of increasing trial number or manipulation salience on this?

Hence there are several steps that researchers can immediately take to produce more reliable, informative and accurately described results from small sample studies. In the longer term, an important goal for comparative cognition is to estimate the natural variation in behavior of animals across sites and times. This will help us to understand how the results of individual studies might generalize to other samples of the same species, and thus the confidence that we can have in the between-species inferences we make when testing evolutionary hypotheses (Klein et al., 2018; ManyPrimates et al., 2019a; Voelkl & Würbel, 2019). Currently, many of these inferred differences between species may be inaccurate as they are made from non-representative samples and are confounded by a host of site-specific factors (Voelkl & Würbel, 2019; Yarkoni, 2019). If the results from individual samples are not reliable, then the differences between multiple samples might not be either. Comparative cognition would therefore benefit from large-scale projects aimed at estimating the magnitude of between site differences in behavior of the same species across a range of research designs, and whether this itself varies across species. These projects will increase the value of *all* comparative cognition research, past, present and future. This is particularly important for research with uncommon or hard-to-reach samples, which will need to generalize from the results of multi-site collaborations with other species to understand the robustness of their effects.

However, these many-site studies should not be seen as unimpeachable. Comparative cognition will falter if it begins to roll-out insensitive studies across a large number of species with a view to understanding the evolution of cognition from single studies with small samples across a range of taxa. It is therefore encouraging that the ManyPrimates collaboration are taking a considered approach towards establishing an infrastructure that will allow them to map the variability of primate behavior (ManyPrimates et al., 2019a, b).

While this paper has argued for an assessment of the replicability of comparative cognition research and has advocated for large-scale collaboration in order to map the variability of animal behavior, it is important not to lose the diversity of our research (Beach, 1950). Studies which increase the amount of resources used (e.g., sample sizes, trials, sites) may wish to build-in more diversity into their study designs and analyses to provide more generalizable results (Baribault et al., 2018; Yarkoni, 2019). In terms of replication studies, choosing which studies will be most important and informative to replicate is a key issue (Coles et al., 2018). For example, a key claim based on weak evidence probably should be replicated before being used as a building block for future research, whereas a study with a known confounding variable might be less helpful to replicate, even if it was historically significant. Finally, it is essential to realize that there will be no one-size-fits all solution to replicability across comparative cognition. Some areas may already know they have few issues, some may be beginning a longer journey to trace which of it results are reliable. Moving forward, comparative cognition should discuss how reliable it wants its research to be. A reliable comparative cognition is not a comparative cognition that always produces *p* < .0001. Such a system could severely restrict the hypothesis-space we can sample. Rather, reliability should be something we can predict and justify as a community given our goals and resource constraints. One solution could be having parallel research streams, one involving large-scale multi-site collaborations mapping the variability of animal performance and providing stronger tests of evolutionary theory, one involving small-scale exploratory research to generate hypotheses about animal cognition, and one providing robust assessments of these hypotheses at the individual-level or group-level but with small sample sizes. Each of these streams should take responsibility for estimating and justifying the reliability of its approach and focus on how it can communicate the uncertainty of the data it generates. However, the optimum reliability and uncertainty of the results might differ between the streams.

Finally, the steps to understand the replicability and reliability of comparative cognition research have large resource, infrastructure and pedagogical requirements. While individual laboratories can contribute by assessing the robustness of their own results, the most effective and long-lasting solutions will require communication and collaboration between laboratories, funding bodies, journals, editors and endorsement by senior researchers. Establishing an infrastructure for a reliable and productive comparative cognition requires a long-term vision. Projects such as ManyPrimates and The Atlas of Comparative Cognition (https://sites.google.com/view/acoco) can help to form a field that can create and combine data necessary to understand how cognition evolves. However, to realize these benefits they require investment to create and maintain them. Such projects will be slow and long-term, and this presents us with a challenge to overcome the current incentives promoting rapid “discovery.” While comparative cognition’s issues with replicability are in many ways yet to be revealed, the finding that many claims might be unsubstantiated represents a unique opportunity for the field to understand and improve itself as a science. For readers who are interested in learning more about replication science we have made a list of suggested introductory reading in Table 3.

## Supplementary Material

Appendix

## Figures and Tables

**Table 1:** The Results of 40,000 Simulated Comparative Cognition Studies by Power.

Power	Proportion Published	Unstandardized Effect Size		
		All Samples	Published	Mean overestimation in “published” findings

80	0.796	5.77	6.53	13%
50	0.494	3.82	5.56	45%
20	0.205	1.89	4.88	158%
5	0.053	0.007	4.45	64486%

*Note*. The proportion published represents the proportion of studies producing p < .05, and the unstandardized effect sizes are the mean differences between the groups.

**Table 2:** The Mathematically Derived Probability of a Successful Replication Attempt of an Original Study Randomly Selected from a Given Range of p-values from the 15,471 "Published" Simulation Studies.


*p*-value original study	*p* ≤ 0.01	0.01 < *p* ≤ 0.02	0.02 < *p* ≤ 0.03	0.03 < *p* ≤ 0.04	0.04 < *p* ≤ 0.05
Probability of successful replication	0.67	0.57	0.52	0.50	0.48


**Table 3:** Recommended Reading to Introduce Readers to Current topics in Replication Research

Title	Reference
Reproducibility of scientific results	Fidler & Wilcox (2018)
Detecting and avoiding likely false-positive findings—A practical guide.	Forstmeier et al. (2017)
Justify your alpha	Lakens et al. (2018)
The regression trap and other pitfalls of replication science	Fiedler & Prager (2018)
Re-thinking reproducibility as a criterion for research quality	Leonelli (2018)

## Footnotes

1 While this paper focuses on statistical significance, as this is the framework that most research is performed in, the quality of statistical and scientific thinking of researchers might be improved by moving beyond significance and focusing on accepting and communicating uncertainty (Gelman, 2017; Gelman & Carlin, 2017; McShane & Gal, 2016; McShane et al., 2019).

2 This was because the existing birds died and it has not been possible to obtain new birds for various practical reasons.

3 These differences in behavior might reflect an adaptive response to many subtle environmental differences between sites, and as such “independent replicate studies that fail to reproduce the original findings might not necessarily indicate that the original study was poorly done or reported, but rather that the replicate study was probing a different region of the norm of reaction” (Voelkl & Würbel, 2019, p. 3). The reaction norm is a concept used by Voelkl and Würbel to represent gene x environment interactions acknowledging both plasticity and canalization.
