# Supplementary material for: Replications in Comparative Cognition: What Should We Expect and How Can We Improve?
Source: Anim Behav Cogn. Author manuscript; Available in PMC 2020 Jul 3. (PMC7334049; doi:10.26451/abc.07.01.02.2020)
Supplement: Appendix [file EMS85731-supplement-Appendix.pdf]

Appendix

Simulation Study 1 Details

The code for the simulations is available at: https://github.com/BGFarrar/P-value-simulations/blob/master/CCreplicationsV1.R).

Data were simulated from two normal distributions for each of the four sets of simulations:

Power	Population 1	Population 2
80%	$X \sim N(50, 5)$	$X \sim N(55.78, 5)$
50%	$X \sim N(50, 5)$	$X \sim N(53.82, 5)$
20%	$X \sim N(50, 5)$	$X \sim N(51.87, 5)$
5%	$X \sim N(50, 5)$	$X \sim N(50, 5)$

The difference between Population 1 and Population 2 was calculated in order to give the desired power for a one-tailed two sample *t*-test with n = 10 per group.

10,000 samples were then taken from each Population and compared to each other, and the *p*-values and mean difference between each sample recorded. The proportion of *p*-values under .05 was calculated, and the mean difference between samples associated with these *p*-values was compared to the mean difference across all samples to calculate the unstandarised effect size inflation.

Next, the expected number of exact replication studies that produced a significant result in the same direction as the original was calculated by multiplying the number of significant results from the simulation by the power of test again, and this was performed for a range of *p* values (Table 2), as well as overall.

Finally, although not included in the manuscript, the exact replication studies were also simulated – predictably, this was consistent with the mathematical derivation, and the *p*-value distributions of the original published studies and the replication studies are given below in Figures A1 and A2.

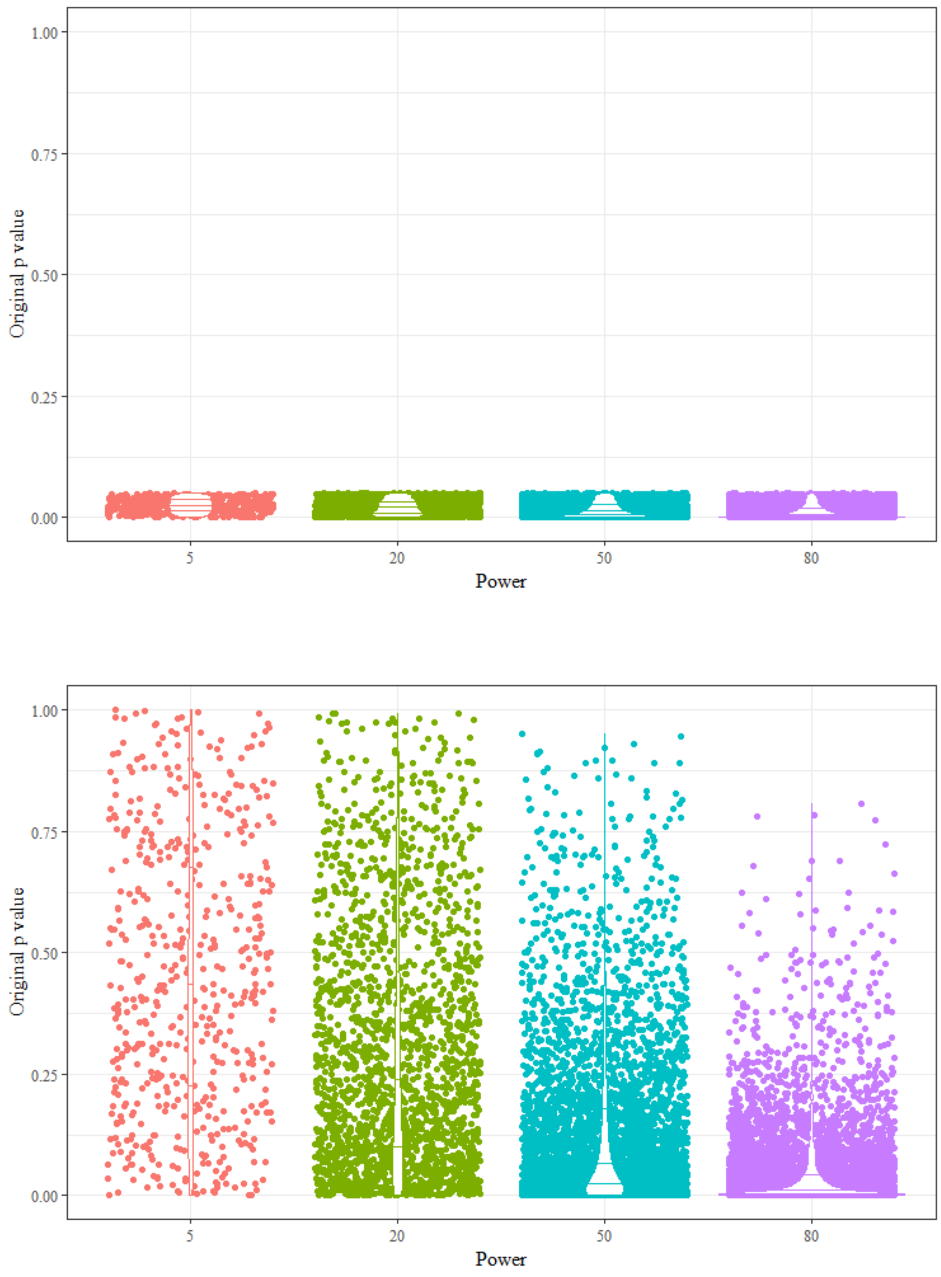

Figure A1. The *p*-value distributions of the original significant studies, by power (top), and the *p*-value distribution of their exact replication studies by power in % (bottom). Violin plots are overlaid displaying the range and quantiles 25, 50 and 75.

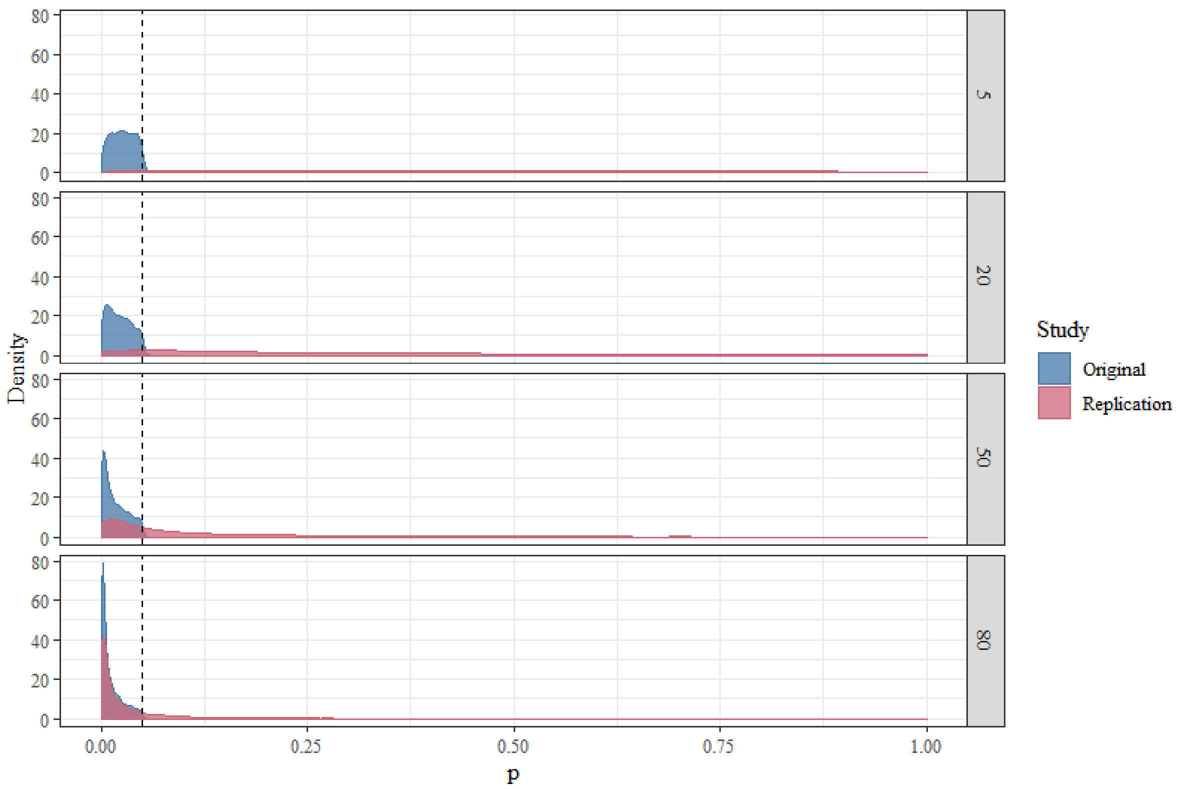

Figure A2. Density distributions of the *p*-values from the first simulation study. Blue plots represent the *p*-value distribution of the original studies, which all fall below .05 due to a simulated publication bias. Red plots represent the *p*-value distribution of the exact replication studies. The four plots are arranged by the power of the studies, from 5% at the uppermost panel, to 20%, 50% and 80% at the lowermost panel.

Simulation Study 2 Details

Again the code for the second simulation can be found at: https://github.com/BGFarrar/P-value-simulations/blob/master/CCreplicationsV1.R

The data were simulated using edited code from DeBruine and Barr (2019). Data were simulated from the following model:

$$LT_{si} = \beta_0 + S_{0s} + I_{0i} + (\beta_1 + S_{1s})X_i + e_{si}$$

This model is identical to DeBruine & Barr (2019, p. 7), but we swapped LT (Looking Time) for RT (Reaction Time). The looking time for subject s on item i, LT_{si}, is composed of a population grand mean β_0, a by-subject random intercept S_{0s}, a by-item (either physically possible or impossible image) random intercept I_{0i}, a fixed slope β_1, a by-subject random slope S_{1s}, and a trial-level residual e_{si}. X_i is the condition.

Across all of our simulations, the following parameters were simulated with the following:

β_0: 1000
S_{0s} ~ N(0, 100)
I_{0i} ~ N(0, 5)
S_{1s} ~ N(0, 40)
e_{si}: 200

Subjects were simulated with a correlation between intercepts and slopes of 0.2, meaning that subjects with larger looking times showed on average larger looking time differences.

Across the simulations we varied the main effect of condition and the number of trials in a 3 x 3 design:

β_1 (200, 100, 0) x trials (1, 5, 100)

10,000 datasets were simulated for each design, and the analyses differed slightly between the designs to avoid singular fits. For the single trial designs, the data were analyzed using paired *t*-tests, and for the five and one hundred trial designs, the data were analyzed using a mixed effect model with the following structure:

lmer(LookingTime ~ Condition + (1 | subj_id), simulateddata, REML = FALSE)

Finally, for the five trial conditions, the calculated *p*-values might be slightly inaccurate as a small proportion of simulations still led to singular fits. This may also be something to consider when interpreting the analysis of Bird and Emery (2010), which has even more parameters in the model.
